# Depression and Anxiety as a Risk Factor for Myocardial Infarction

**DOI:** 10.7759/cureus.6064

**Published:** 2019-11-03

**Authors:** Kheraj Mal, Inayatullah D Awan, Jaghat Ram, Faizan Shaukat

**Affiliations:** 1 Cardiology, National Institute of Cardiovascular System, Sukkur, PAK; 2 Psychiatry, Ghulam Muhammad Mahar Medical College, Sukkur, PAK; 3 Cardiology, Ghulam Muhammad Mahar Medical College, Sukkur, PAK; 4 Internal Medicine, Jinnah Postgraduate Medical Centre, Karachi, PAK

**Keywords:** myocardial infarction, depression, anxiety, risk factors

## Abstract

Introduction: Patients having a cardiovascular disease experience negative states of psychology. An increased incidence of coronary artery disease is attributed to both depression and anxiety.

Materials and methods: In this retrospective study, the Hospitalized Anxiety and Depression Scale (HADS) was used to determine anxiety and depression in stable patients of myocardial infarction (MI) at the time of their discharge. All responses were based on the patients’ perceptions two weeks prior to acute MI event. SPSS version 21.0 was used for data entry and analysis.

Results: The mean age of the participants in our study was 49.09±5.61 years. About 52.83% (n=28) and 58.49% (n=31) participants suffered from anxiety and depression two weeks prior to their myocardial infarction.

Conclusion: Depression and anxiety can be a risk factor for myocardial infarction in susceptible individuals. Attention should be given to mental well-being, and a multi-disciplinary management approach should be taken for these patients including psychiatry and psychology.

## Introduction

Patients with cardiovascular disease (CVD) experience psychological states that are usually negative. An increased incidence of coronary artery disease is attributed to both depression and anxiety [1]. The occurrence and sometimes the progression of CVD are due to anxiety. Coronary artery disease (CAD) can also develop due to anxiety in patients without existing CVD. As reported by Roest and colleagues in a meta-analysis, including 250,000 subjects and 20 studies, anxiety increases the risk of CAD occurrence by 26% along with other medical factors [2]. Along with this, CVD can also be developed by depression in people who are initially healthy [3]. The risk of the onset of CAD is 1.64 times more in patients with symptoms of depression [4].

Despite the fact that there were significant overall findings, only 10 out of 20 studies report a significant association between anxiety and the occurrence of CAD in multivariate analyses. This means that there can be a heterogeneous link between these two mental health issues and myocardial infarction [1]. Also, inconsistent outcomes have been reported by studies on depression being a risk factor for myocardial infarction (MI) [5]. To our knowledge, Pakistan does not have any data available that examines the impact of psychological symptoms like depression and anxiety in acute MI. Therefore, this study is being performed, keeping in view the above gaps in knowledge.

## Materials and methods

This retrospective study was conducted in the National Institute of Cardiovascular Disease, Sukkur, from 1st Jan to 30th Sept 2019. All participants of both genders admitted with MI who were stable at the time of discharge were included in this study after informed consent. The institutional review board approved the study.

During the study period, 222 patients were admitted with MI. There were 27 mortalities, 58 were excluded as they were smoker, diabetic, hypertensive, or obese (body mass index (BMI) >30 kg/m^2^), and 84 patients did not consent to participate. The remaining 53 participants were included in the study.

Hospitalized Anxiety and Depression Scale (HADS) was administered to all participants. All responses were based on the feelings of the patients two weeks prior to their MI. HADS has robust psychometric properties and is brief and easy to administer. It consists of 14 items divided into two 21-point subscales for anxiety and depression, and a score of ≥8 was considered to be abnormal for either anxiety or depression [6].

Data were analyzed using SPSS Version 21.0 (IBM Corp., Armonk, NY). Mean, and standard deviation (SD) was calculated for continuous variables such as age and HADS score. Frequencies and percentages were calculated for categorical variables, including gender, type of MI, and HADS score.

## Results

The mean age of participants in our study was 49.1 ± 5.6 years. There were more men than women (58% vs. 42%). ST elevated MI (STEMI) was more common (55%). As many as 53% suffered from anxiety and 59% from depression two weeks prior to their MI episode (Table 1).

**Table 1:** Demographics and Frequencies of Anxiety and Depression Abbreviations: HADS, Hospitalized Anxiety and Depression Scale; NSTEMI, Non ST Elevated Myocardial Infarction; SD, Standard Deviation; STEMI, ST Elevated Myocardial Infarction.

Patient Characteristics	Frequency (%)
Age	49.1 ±5.6
Gender	
Male	31 (58.5%)
Female	22 (41.5%)
Types Myocardial Infarction	
STEMI	29 (54.7%)
NSTEMI	24 (45.3%)
HADS Score	
Anxiety (Mean ± SD)	6.9 ± 4.6
Depression (Mean ± SD)	7.2 ± 4.8
Participants with Anxiety	28 (52.8%)
Participants with Depression	31 (58.5%)

## Discussion

A complex association exists between anxiety and cardiovascular (CV) health; therefore, a comparison to assess how strong a relationship exists between anxiety and cardiovascular disease (CVD) occurrence along with other psychological elements is highly needed. There was a 46% increased risk of CVD due to depression, as reported by Van der Kooy et al. [7]. Another study conducted by Roest et al. showed a 55% increased risk of cardiac death in patients with depression that was comparable to the effect of anxiety [2]. Several mechanisms might explain the adverse relation between anxiety and heart disease. In various studies, anxiety has been linked with atherosclerosis, decreased heart rate, and risk of ventricular arrhythmias [8-10].

The results of our study show that 52.83% of patients had anxiety two weeks before they had myocardial infarction. Anxiety can be a usual reaction to a tense or stressful situation, e.g., an acute cardiac event. However, if anxiety propels patients to get more involved in treatment (e.g., daily workout, adherence to therapy), it can be useful for the patients. But if anxiety persists for long periods of time or if it is in excess, then it can be harmful for psychological health and the overall well-being of an individual [4]. The risk of the incidence of CVD increases by 26% in anxious people, as shown by Roest et al. [2]. The risk of cardiac mortality also specifically increases due to anxiety, as there is a 48% increase in the risk of cardiac deaths in anxious persons. There also has been a relationship between anxiety and the occurrence of non-fatal MI [2].

As already discussed, 58.5% of patients had depression just two weeks before they had their MI attack. Depression is linked to an elevated risk of chronic diseases that cover chronic heart disease (CHD) as well, as shown by a large number of epidemiological studies during the last 10 years. Depression has found to be an independent risk factor for CVD, apart from the conventional known risk factors that include obesity, smoking, diabetes, sedentary lifestyle, and hypertension, as reported by Meng et al. [11]. Moreover, the negative impact of depression over cardiac parameters can be triggered by both biological and behavioral factors. The major behavioral factors include smoking, non-compliance with cardiac medications, and physical inactivity [12].

The study was first of its kind to evaluate study depression and anxiety as a risk factor for myocardial infarction in our demographic region. We tried to eliminate confounding factors by excluding smokers, diabetic, hypertensive, and obese patients. However, it has its limitations. First, it was a single-center study; hence, the results cannot be projected to a broader population. Second, the sample size was small. Third, since it was a retrospective study, patients may not remember their symptoms, which may have caused bias in calculating depression and anxiety scores. Despite our best effort, there were still a few confounding factors such as previous history of MI, family history, and lack of physical exercise. There was a high rate of non-consent (84/222; 37.8%), which indicates the resistance of the population towards discussing mental health issues and psychological well-being. Further large scale prospective trials are needed to understand and establish the definite role of anxiety and depression in myocardial infarction.

## Conclusions

In conclusion, this study suggests depression and anxiety are significantly associated with an increased risk of myocardial infarction. Because anxiety, depression, and myocardial infarction are highly prevalent, they have significant implications for public health. Prevention and treatment of depression and anxiety may reduce myocardial infarction. 
